# M-type channels selectively control bursting in rat dopaminergic neurons

**DOI:** 10.1111/j.1460-9568.2010.07107.x

**Published:** 2010-03

**Authors:** Guillaume Drion, Maxime Bonjean, Olivier Waroux, Jacqueline Scuvée-Moreau, Jean-François Liégeois, Terrence J Sejnowski, Rodolphe Sepulchre, Vincent Seutin

**Affiliations:** 1 Laboratory of Pharmacology and GIGA Neurosciences, University of LiègeBelgium; 2 Systems and Modeling, Department of Electricity, Electronics and Computer Science, University of LiègeBelgium; 3 Howard Hughes Medical Institute, The Salk Institute for Biological Studies and Department of Biological Sciences, The University of CaliforniaSan Diego, CA, USA; 4 Laboratory of Medicinal Chemistry and Drug Research Center, University of LiègeBelgium

**Keywords:** firing patterns, M-current, modeling, slow pacemakers, small conductance Ca^2+^-activated K^+^-channels, substantia nigra

## Abstract

Midbrain dopaminergic neurons in the substantia nigra, pars compacta and ventral tegmental area are critically important in many physiological functions. These neurons exhibit firing patterns that include tonic slow pacemaking, irregular firing and bursting, and the amount of dopamine that is present in the synaptic cleft is much increased during bursting. The mechanisms responsible for the switch between these spiking patterns remain unclear. Using both *in-vivo* recordings combined with microiontophoretic or intraperitoneal drug applications and *in-vitro* experiments, we have found that M-type channels, which are present in midbrain dopaminergic cells, modulate the firing during bursting without affecting the background low-frequency pacemaker firing. Thus, a selective blocker of these channels, 10,10-bis(4-pyridinylmethyl)-9(10*H*)-anthracenone dihydrochloride, specifically potentiated burst firing. Computer modeling of the dopamine neuron confirmed the possibility of a differential influence of M-type channels on excitability during various firing patterns. Therefore, these channels may provide a novel target for the treatment of dopamine-related diseases, including Parkinson’s disease and drug addiction. Moreover, our results demonstrate that the influence of M-type channels on the excitability of these slow pacemaker neurons is conditional upon their firing pattern.

## Introduction

Midbrain dopaminergic (DA) neurons sustain important physiological functions such as control of motricity and signaling of positive error in reward prediction in the mesolimbic system (Schultz, 2007). A dysfunction of the DA system is implicated in the pathophysiology of Parkinson’s disease, schizophrenia and drug abuse (Iversen & Iversen, 2007). Under physiological conditions, DA neurons can switch between three distinct modes: tonic (‘pacemaker’), irregular and burst firing (Grace & Bunney, 1984; Brazhnik *et al.*, 2008). Low-frequency pacemaking of DA neurons mainly involves voltage-dependent Ca^2+^ channels (Puopolo *et al.*, 2007). Because burst firing increases synaptic concentrations of dopamine (Chergui *et al.*, 1994), many studies have focused on the factors controlling the switch to this firing pattern. It is generally agreed (see Overton & Clark, 1997) that bursting requires a glutamatergic input stimulating *N*-methyl-d-aspartate (NMDA) receptors. This has been further demonstrated recently by the observation that selective genetic inactivation of NMDA receptors in these neurons strongly disrupts burst firing, with many important behavioral consequences (Zweifel *et al.*, 2009). However, activation of GABA_A_ receptors inhibits bursting because of their shunting effect on the oscillatory behavior (Tepper & Lee, 2007). Finally, both *in-vitro* and *in-vivo* experiments show that a reduction of a potassium conductance mediated by small conductance Ca^2+^-activated K^+^ (SK) channels greatly potentiates irregularity and/or bursting (Shepard & Bunney, 1988, 1991; Nedergaard *et al.*, 1993; Seutin *et al.*, 1993; Waroux *et al.*, 2005; Ji & Shepard, 2006).

We and others recently described the presence of another K^+^ current in DA neurons (Hansen *et al.*, 2006; Koyama & Appel, 2006). This current had the typical electrophysiological signature (Brown & Adams, 1980; Kuffler & Sejnowski, 1983) and pharmacology of the M-current, being enhanced by retigabine and blocked by 10,10-bis(4-pyridinylmethyl)-9(10*H*)-anthracenone dihydrochloride (XE991) (Wang *et al.*, 1998; Tatulian *et al.*, 2001). Moreover, one of the subunits carrying M-currents (KCNQ4) was highly expressed in DA neurons (Hansen *et al.*, 2006). However, blockade of the M-current by XE991 had only minor effects on the spontaneous firing of DA neurons in rat brain slices and *in vivo* (Hansen *et al.*, 2006; and see Results). Thus, the M-current does not appear to act as a ‘brake’ on low-frequency firing. We hypothesized that, given its voltage dependence, its rather slow activation rate and lack of inactivation, the M-current could be specifically involved in controlling the bursting behavior in these cells. Indeed, depolarized plateaus are observed in DA neurons during bursting (Grace & Bunney, 1984) and they are *a-priori* sufficiently long-lasting (200–700 ms) to enable activation of M-channels. We tested this hypothesis using a combination of *in-vivo* extracellular recordings of nigral DA neurons, intracellular recordings in a brain slice preparation and computer modeling in which M-channels were added to a published model of DA neurons (Canavier & Landry, 2006).

## Materials and methods

All procedures were carried out in accordance with guidelines of the European Communities Council Directive of 24 November 1986 (86/609/EEC) and were accepted by the Ethics Committee for Animal Use of the University of Liège (protocol 86).

### In-vivo experiments

#### Housing

Adult male Wistar rats were housed in groups of three or four, supplied with food and water *ad libitum*, and maintained on a 12 h light/dark cycle.

#### Recordings

Rats (200–250 g) were anesthetized with chloral hydrate (400 mg/kg, i.p.). Additional supplemental doses were injected intraperitoneally when necessary. Their temperature was maintained at 36–37°C by means of a heating pad. The rats were placed in a stereotaxic apparatus (Model 902, Kopf). After removing a small part of the skull between the lambda and bregma, above the implantation point, the tip of the pipette was lowered into the brain at the following coordinates (with the lambda as reference) for the substantia nigra pars compacta: 2 mm anterior, 1.8–2.2 mm lateral and 6–7 mm under the cortical surface, depending on the location in the frontal plane.

#### Electrodes and iontophoresis

All electrodes were made as described previously (Waroux *et al.*, 2005) and consisted of a recording electrode and a five-barrel iontophoresis pipette glued together. For most experiments, each barrel was filled with one of the following solutions (dissolved in 30 mm NaCl): GABA (100 mm, pH 4), 30 mm NaCl (control solution in some experiments), *N*-methyl-laudanosine (NML) (10 mm, pH 7), 2-(3-carboxypropyl)-3-amino-6-(4-methoxyphenyl) pyridazinium bromide (SR95531) (1–10 mm, pH 7) or XE991 (1 mm, pH 7), NaCl (0.5 m, current balancing), and, in some experiments, dopamine (100 mm, pH 7). The iontophoretic pipette was broken back at the tip to a diameter of approximately 20–30 μm. The recording electrode had a tip diameter of 1–3 μm and a resistance of 8–10 MΩ when filled with 0.9% NaCl. A negative retention current of −10 nA was used between ejection periods. Drugs were ejected for 5 min, unless stated otherwise.

#### Action potential recordings and identification of neurons

Action potentials (amplitude: 200–1000 μV) were passed through an impedance adapter and amplified 1000 times with a home-made amplifier. They were displayed on an oscilloscope and fed to an analog–digital interface (CED 1401) connected to a computer. Data were collected with the use of the ‘spike 2’ software (Cambridge Electronic Design, Cambridge, UK). Several scripts were used to analyse firing patterns and various characteristics of bursts.

Electrophysiological and pharmacological parameters were used in order to identify DA neurons as described previously (Waroux *et al.*, 2005). *In vivo*, these neurons exhibit an irregular firing pattern with interspersed bursting episodes and long (> 2.5 ms), triphasic spikes (with a positive first phase), often displaying a prominent notch in the initial positive rising phase. They have a slow firing rate of between 0.5 and 5 Hz. In some experiments, dopamine was iontophoresed as pharmacological control and always induced a slowing or complete cessation of the firing of the neurons, as expected.

#### Data analysis

To quantify the effect of SK-channel blockers *in vivo*, we used established criteria of irregularity measurement of DA neuron firing *in-vivo* (Grace & Bunney, 1984; Freeman *et al.*, 1985). We quantified the number of spikes generated in bursts as a percentage of all spikes within a given period (1 min). Parameters defining burst firing were at least three successive spikes with a maximal interspike interval (ISI) of 80 ms between the two-first spikes and a maximum of 160 ms for all intraburst ISIs. Only experiments in which all data could be obtained were selected for further analysis, except for one intraperitoneal experiment in which a very high percentage of spikes in bursts was observed during XE991 with 30 nA NML (97.21%) before losing the cell and in which the same value was extrapolated for 60 and 90 nA. Experiments in which the firing rate differed by more than 25% between the first control period (before NML) and the end of the wash-out period of NML (before applying XE991) were also rejected.

### Slice experiments

Methods were as described previously (Scuvée-Moreau *et al.*, 2004). Briefly, male Wistar rats (150–200 g) were anesthetized with chloral hydrate (400 mg/kg, i.p.) and decapitated. The brain was rapidly removed and placed in cold (∼4°C) artificial cerebrospinal fluid of the following composition (in mm): 126 NaCl, 2.5 KCl, 1.2 NaH_2_PO_4_, 1.2 MgCl_2_, 2.4 CaCl_2_, 11 glucose, 18 NaHCO_3_, saturated with 95% O_2_ and 5% CO_2_ (pH 7.4). A block of tissue containing the midbrain was placed in a Vibratome (Coretech, St. Louis, MO, USA) filled with the same solution and cut in horizontal slices (thickness: 350 μm). The slice containing the substantia nigra pars compacta was completely immersed in a continuously flowing (∼2 mL/min), heated solution (34 ± 0.5 °C) of the same composition as indicated above. Intracellular recordings were made using glass microelectrodes filled with 2 m KCl (resistance: 70–150 MΩ). All recordings were made in the bridge-balance mode, using a BA-1S amplifier (NPI Electronic GmbH, Tamm, Germany). Membrane potentials and injected currents were recorded on a TA240 chart recorder (Gould Instrument Systems, Valley View, OH, USA) and a Combiscope oscilloscope (Fluke Corp., Everett, WA, USA). The Flukeview software was used for off-line analysis.

The DA neurons had a slow (0.5–4 Hz) spontaneous firing rate, broad action potentials, a large I_h_ current and a prominent afterhyperpolarization (Scuvée-Moreau *et al.*, 2002).

### Statistics

Because a Shapiro-Wilk test showed that some *in-vivo* data were not normally distributed, these experiments were analyzed with a non-parametric test (Wilcoxon test for paired values). The slice experiments were analyzed with a repeated-measures anova test, followed by a *post-hoc* Newman Keuls test. Simulation data were analyzed using Student’s *t*-tests for paired values. The level of significance was set at *P* < 0.05 in all cases. All statistical analyses were run on statistica° (version 8) (StatSoft, Tulsa, OK, USA). For clarity, all of the results are expressed as means ± SEM.

### Drugs

The sources of the drugs used were as follows. Apamin, dopamine and GABA were obtained from Sigma (St Louis, MO, USA). NML was synthesized and evaluated in our laboratory (Scuvée-Moreau *et al.*, 2002). XE991, CGP55845, 6-cyano-7-nitro-quinoxaline-2,3-dione (CNQX) and 2-amino-5-phosphonopentanoic acid (APV) were purchased from Tocris Cookson (Bristol, UK). The GABA_A_ antagonist SR95531 was a gift from Sanofi (Paris, France).

### Computational methods

The computational model of a single DA neuron was based on Canavier & Landry (2006) with some modifications (Bonjean *et al.*, 2007). The model included a soma, four branched proximal and eight distal dendrites.

All compartments were capable of generating action potentials and contained a fast sodium current *I*_Na_, a delayed rectifier potassium current *I*_K,dr_, a transient outward potassium current *I*_K,A_, a leak current *I*_leak_ and a Na^+^/K^+^ pump, as well as sodium dynamics and a sodium balance ruled by the following equations


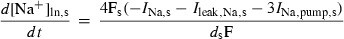



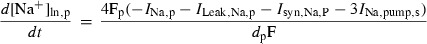



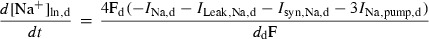


where F is the Faraday constant, the subscripts s, p and d denote the compartment (soma, proximal and distal) and *I*_syn_ is the sum of synaptic currents mediated by Na^+^, i.e.


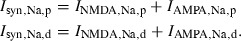


The soma contained a hyperpolarization-activated cation current *I*_H_ and a SK K^+^-current activated by calcium. Calcium entered the soma through voltage-activated T-type, N-type and L-type currents and calcium was removed with a calcium pump. M-type channels, producing the *I*_K,M_ current (M-current), were also included in the somatic compartment (Bonjean *et al.*, 2007), with the following dynamics


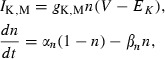


where the channel opening and closing rate constants α_*n*_ and β_*n*_ are defined by (Bibbig *et al.*, 2001)


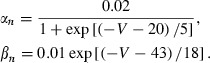


The maximal conductance of the M-current was set to 300 μS/cm^2^, giving a conductance of 196 μS/cm^2^ at −45 mV. This is approximately five times the conductance that can be calculated from published data (36 μS/cm^2^; Koyama & Appel, 2006), which were obtained using dissociated DA neurons. When we used the value of the conductance found experimentally, the effect was qualitatively similar but much less robust (see Results). Possible reasons for this discrepancy include an underestimation of the conductance in the experiments because of damage to the channels produced by the dissociation procedure or because of a higher current density in the dendrites vs. the soma. Alternatively, some fine adjustments of the model may be needed. Indeed, many of the parameters that have been implemented in the model have not been directly measured in DA neurons but have usually been taken from published data on other types of neurons. Moreover, other features of DA neurons (presumably the high density of Na_v_ channels in the axonal initial segment, axon originating from a primary dendrite in many DA neurons) have not been taken into account. The development of a completely accurate model will therefore only be possible when all of this information is available.

Synaptic AMPA and NMDA receptors, mediating *I*_AMPA_ and *I*_NMDA_ currents, were located on the dendritic compartments. GABA_A_ receptors, mediating the *I*_GABA,A_ current, were located on both somatic and dendritic compartments with a density ratio of 1 : 10.

All currents followed a Hodgkin-Huxley kinetic scheme (Hodgkin & Huxley, 1952). The dynamics of the synaptic currents were modeled with a two-state kinetic scheme (Destexhe *et al.*, 1994). Glutamatergic synaptic events were generated by a Poisson stochastic process, which mimicked *in-vivo*-like activity (Canavier & Landry, 2006).

Simulations were performed under the NEURON modeling program, with a Runge-Kutta fourth-order integration method, on a Pentium 4 3 GHz. Analyses of computational data were carried out with MATLAB 7 (R14).

The mean charge transfer through M-channels in each condition was calculated by taking the mean values of the simulated M-current in the model for six simulations. The M-current density before action potentials was obtained by measuring the instantaneous values of the M-current at 20 ms before the onset of action potentials (which was defined as the crossing of −40 mV during its ascending phase). These values were obtained before 260 events in control conditions and 350 events during I_K,SK_ inhibition.

**Fig. 3:**
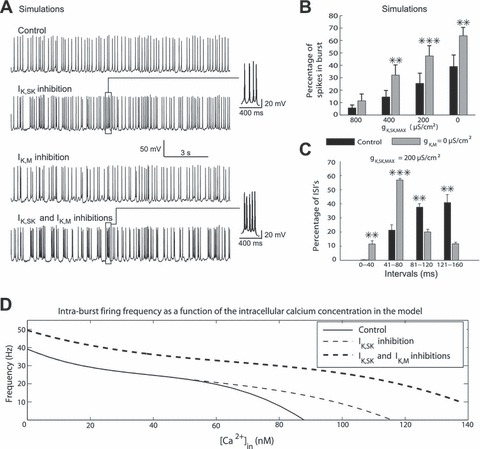
Simulations of M-current blockade on a DA neuron model confirm its selective effects on burst firing. (A) Example of a simulation. M-current inhibition was modeled by setting the M-current conductance to 0, whereas the effect of NML was modeled by reducing the I_K,SK_ conductance (in this case to 0). The temporal pattern of synaptic inputs was exactly the same in the four traces. (B) Simulations (*n* = 6) showed a significant potentiation of bursting of the model DA neuron during M-current inhibition. (C) A significant shift of ISIs toward the shorter intervals is seen in the model, as also illustrated by the insets in (A). (D) Effect of I_K,SK_ and combined I_K,SK_ and I_K,M_ inhibitions on the relationship between [Ca^2+^]_in_ and intraburst firing rate. **P < 0.01; ***P < 0.001.

In a few simulations (Fig. 3D), the cytoplasmic calcium concentration [Ca^2+^]_in_ in the model was varied and the firing frequency of the simulated trace was computed for each of these values. The intraburst firing frequency was subsequently plotted as a function of the calcium concentration. These particular simulations were performed in the absence of synaptic afferents and while [Na^+^]_in_ was fixed at 3.8, 2.8 and 4 mm for the soma, proximal dendrites and distal dendrites, respectively.

## Results

### Effect of systemic and local application of XE991 on the firing of dopaminergic neurons

In a first series of experiments, we studied the impact of intraperitoneally administered XE991 (3 mg/kg, i.p.) on the firing of DA neurons. In previous *in-vitro* experiments, we had demonstrated that this compound is a specific blocker of the M-current in these cells; thus, it had no effect on the shape of action potentials, the resting membrane potential or the medium-duration afterhyperpolarization induced by the opening of SK-channels (Hansen *et al.*, 2006).

**Fig. 1:**
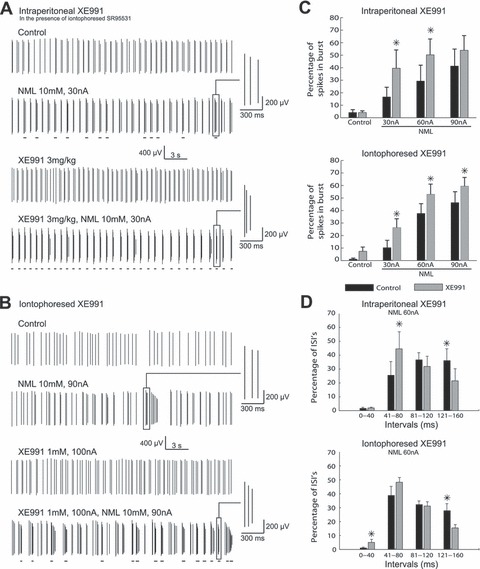
XE991 selectively enhances burst firing of DA neurons *in vivo* and increases the proportion of short ISIs in bursts. (A) M-current blockade was induced by a systemic administration of XE991 (3 mg/kg, i.p.). A GABA_A_ antagonist (SR95531) was also iontophoresed (1 mm, 100 nA) during these recordings to block the most important inhibitory afferences. (B) Local M-channel blockade was performed by iontophoresis of XE991 (1 mm, 100 nA). Events that are underlined correspond to bursts (see Materials and methods for our criteria). (C) Histogram showing a significant potentiation of bursting of DA neurons during M-current blockade (intraperitoneal XE991, *n* = 6; iontophoresed XE991, *n* = 8). (D) Both intraperitoneal and local XE991 applications induced a significant shift of ISIs toward the shorter intervals, as also illustrated by the insets in (A) and (B). *P < 0.05.

The effect of intraperitoneal XE991 was variable, ranging from no change to a large inhibitory effect (*n* = 6) (Fig. S1). This could be due to a mixture of direct and indirect factors, as KCNQ channels are also expressed by many neurons that project to DA neurons. The firing of rat DA neurons is under the inhibitory control of GABA_A_ receptors (Tepper & Lee, 2007). The experiments were therefore repeated while iontophoresing a pharmacologically active (Table S1) amount of the specific GABA_A_ antagonist SR95531. Under these conditions, XE991 had no effect on the spontaneous firing rate or pattern (Fig. 1A) of DA neurons; firing rates were 2.9 ± 0.4 and 3.1 ± 0.4 spikes/s (mean ± SEM) (*P* = 0.25, Wilcoxon test, *n* = 6) in control conditions and in the presence of XE991, respectively. The percentage of spikes in bursts (see Materials and methods) was 4.2 ± 2.3 and 7.1 ± 3.7%, respectively (*P* = 0.89, Wilcoxon test, *n* = 6).

To evaluate the ability of the neurons to fire in bursts, we iontophoresed a reversibly acting blocker of SK-channels, NML (10 mm) (Scuvée-Moreau *et al.*, 2002). This procedure facilitates bursting in these cells in an intensity-dependent manner (Waroux *et al.*, 2005), i.e. the percentage of spikes fired in bursts increases as a function of current intensity (30, 60 or 90 nA).

In order to assess the physiological relevance of our model of bursting, we compared the characteristics of natural (i.e. spontaneous) and NML-induced bursts in the absence of any other pharmacological agent. They were found to be remarkably similar (Figs S2 and S3; see also Waroux *et al.*, 2005). Thus, the mean value of the ISIs was similar in both cases, as was the fact that the first ISI was shorter than the next ISIs, the values of which were close to 100 ms. Burst size histograms showed that the relative frequency of the bursts as a function of their number of spikes was similar in both conditions (Fig. S3A). Importantly, a progressive decrease in the amplitude of the extracellularly recorded action potentials was observed in both natural and NML-induced bursts, and this decrease had a mean amplitude (∼20%, Fig. S3B) that was similar in both conditions. The latter data strongly suggest that the membrane potential changes underlying both types of bursts are quantitatively similar, confirming the validity of our model. Moreover, all of these parameters were similar to those described previously (e.g. Grace & Bunney, 1984).

We next examined the influence of XE991 on NML-induced bursts. Figure 1A shows that intraperitoneally administered XE991 potentiated NML-induced bursting when GABA_A_ receptors of DA neurons were blocked with SR95531. The amplitude of the effect of the M-channel blocker was dependent on the NML iontophoresis current intensity. Thus, during 30 nA NML, XE991 increased the percentage of spikes in bursts from 16 ± 8 to 39 ± 15% (*P* = 0.028, Wilcoxon test, *n* = 6). A similar effect was seen during 60 nA (from 29 ± 13 to 50 ± 13%, *P* = 0.028, Wilcoxon test). At 90 nA, no significant effect was observed (from 41 ± 14 to 54 ± 12%, *P* = 0.17, Wilcoxon test), probably because of a saturation effect. Control intraperitoneal injections of the vehicle had no discernible effect (*n* = 3, Fig. S4A).

In order to test whether XE991 acts directly on DA neurons, we next iontophoresed it onto the recorded neurons. For these experiments, we chose not to use a GABA_A_ antagonist in order to mimic as closely as possible the physiological situation. Moreover, local application of XE991 made any indirect effect of the drug unlikely. As shown in Fig. 1B, the effects of the drug were quite similar to those observed after intraperitoneal injection. Thus, XE991 (100 nA) had no effect on tonic firing (3.4 ± 0.6 and 3.5 ± 0.6 spikes/s in control conditions and in the presence of XE991, respectively, *P* = 0.21, Wilcoxon test, *n* = 8) but increased the percentage of spikes in bursts from 10 ± 8 to 26 ± 9% during 30 nA NML (*P* = 0.018, Wilcoxon test) and from 37 ± 10 to 53 ± 11% during 60 nA (*P* = 0.018, Wilcoxon test). At 90 nA, the increase (46 ± 12 to 59 ± 9%) was also significant (*P* = 0.049, Wilcoxon test) (Fig. 1C, lower panel). Iontophoresis of the vehicle had no effect at any intensity of NML iontophoresis (*n* = 5, Fig. S4B).

A close inspection of the bursting behavior revealed that the M-channel blocker also modified it qualitatively. For example, Fig. 1D shows that, when administered either intraperitoneally or by iontophoresis, it increased the proportion of short ISIs during 60 nA (other results are shown in Fig. S5). This is also apparent in the insets of Fig. 1A and B.

### XE991 facilitates fast firing induced by current injection *in vitro*

**Fig. 2:**
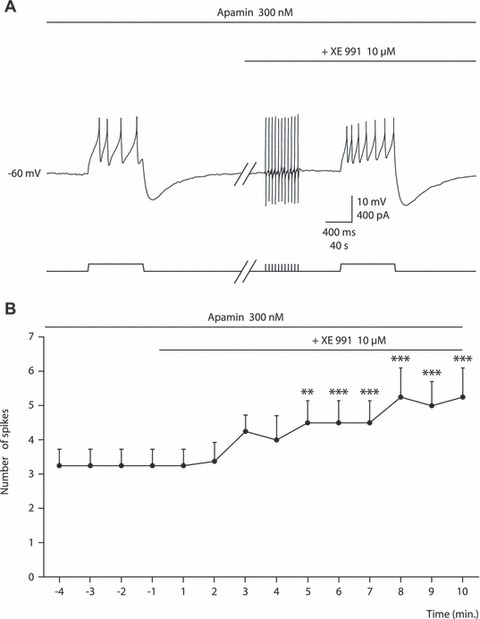
XE991 increases fast firing in DA neurons *in vitro*. (A) Intracellular recording showing that a given amount of current (+ 120 pA) elicits more spikes (truncated in the figure) in the presence of XE991. The experiment was performed in the presence of 300 nm apamin, 10 μm CNQX, 50 μm APV, 10 μm SR95531 and 1 μm CGP55845. Baseline membrane potential was set at −60 mV by a continuous injection of −100 pA. The speed of the recording was reduced 100-fold at the beginning of the superfusion of XE991. (B) Summary plot showing the time-course of the effect of XE991 (*n* = 4). **P < 0.01; ***P < 0.001 vs. control.

In order to confirm the ability of XE991 to facilitate the occurrence of short ISIs in DA neurons, we performed intracellular recordings of these neurons in slices containing the substantia nigra pars compacta. This recording mode was chosen because it is the least likely to disrupt intracellular pathways, which are critical in the control of M-channels (Delmas & Brown, 2005). For these experiments, we superfused the slices with blockers of synaptic transmission (10 μm CNQX, 50 μm APV, 10 μm SR95531 and 1 μm CGP55845) in order to exclude indirect effects. A supramaximal concentration of the SK blocker apamin (300 nm) was used to mimic our *in-vivo* conditions. Neurons were hyperpolarized to −60 mV by negative current injection (−50 to −150 pA) and depolarizing pulses (50–150 pA, 800 ms) were given repeatedly to evoke spikes. In these conditions, 10 μm XE991 significantly increased the number of spikes from 3.2 ± 0.5 to 5.2 ± 0.9 after 10 min (Fig. 2) (*n* = 4) [*F* = 17.1, *P* = 0.001, repeated-measures anova; values after the 5th min of XE991 application were significantly different from those of the control condition (Newman–Keuls test); see Fig. 2 for the various levels of significance]. However, it had no effect on the baseline voltage. XE991 had no significant effect in the absence of apamin (not shown, data from Hansen *et al.*, 2006).

### Computer modeling of the effect of the M-current

We next explored the mechanism underlying our *in-vivo* observations in a model of a DA neuron (Canavier & Landry, 2006), which included amongst others an SK-current and an M-current (see Materials and methods). The activation of synaptic currents was modeled by a Poisson process. Many of the electrophysiological features observed in DA neurons, including low-frequency pacemaker activity and burst firing, were reproduced in the model, which confirmed that the absence of M-current potentiates SK blockade-induced bursting (Fig. 3A). Quantitative analysis of six different model neurons (using different synaptic input patterns) reproduced the experimental results (Fig. 3B, Fig. S5D). This effect was robust when the M-conductance was five times that measured experimentally (Koyama & Appel, 2006; see Materials and methods). When the conductance value was identical to the measured value, the effect was very modest (Fig. S6).

The model also confirmed the ability of M-current blockade to increase the proportion of short ISIs within bursts (Fig. 3C). A plot of intraburst firing frequency vs. [Ca^2+^]_in_ (Fig. 3D) shows the predicted effect of the SK- and M-conductances on the firing behavior of the model neuron. As compared with the control condition, SK blockade allowed faster firing at intermediate [Ca^2+^]_in_ values. Additional block of M-channels shifted the curve to even higher frequencies (as observed experimentally by a higher proportion of short ISIs).

**Fig. 4:**
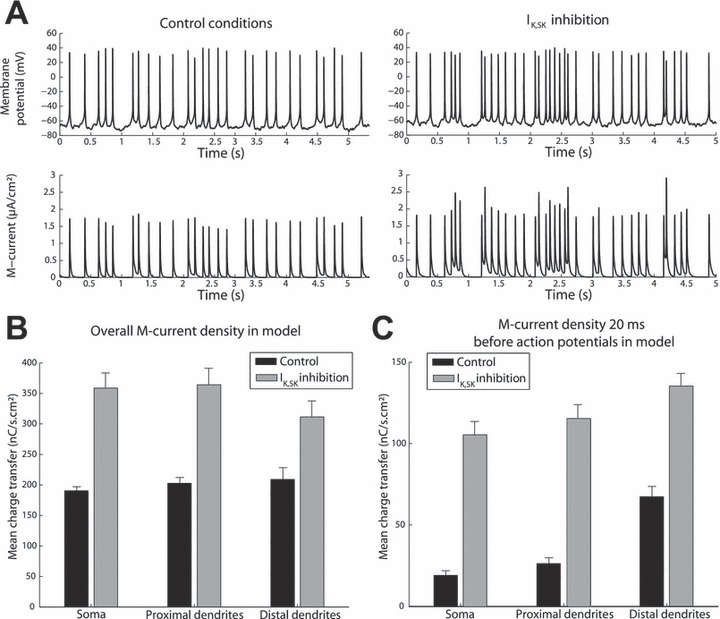
Charge transfer through M-channels in the model when SK-channels are present or absent. (A) Values of the membrane potential (top) and the amplitude of the M-current (bottom) in control conditions (left) and during a I_K,SK_ inhibition (right). (B) Overall M-current density in model. (C) M-current density at 20 ms before action potentials in the model (*n* > 200 events in six modeled cells). The overall M-current density during I_K,SK_ inhibition is only twice that in control conditions. However, the amount of M-current that opposes the generation of action potentials is much larger during I_K,SK_ inhibition. *P* < 0.001 between control and I_K,SK_ inhibition. Student’s t-test for paired values (B) and Student’s *t*-test for unpaired values (C).

We next analyzed the M-current quantitatively in the model, both when the SK-conductance was maximal and when it was set to 0. The charge transfer through the M-conductance was higher in the second condition (Fig. 4A and B). The difference was even more striking when considering the mean charge transfer at 20 ms before the onset of action potentials in both conditions (Fig. 4C; see Materials and methods). Clearly, the M-current completely deactivates between two successive action potentials during low-frequency pacemaker or irregular firing but not when the membrane potential is more depolarized during SK blockade (Fig. 4A).

## Discussion

Taken together, the experimental and modeling data demonstrate that the M-current selectively gates the bursting behavior in DA neurons. The effect that we observe experimentally *in vivo* is most probably due to the blockade of somato-dendritic M-channels, whose existence has been demonstrated experimentally (see Introduction). Indeed, the burst-enhancing effect is observed when GABA_A_ receptors (the major substrate of afferent inhibition in the rat; Tepper & Lee, 2007) are blocked. Furthermore, our slice experiments confirm that XE991 facilitates fast firing in these neurons by a direct effect. Although the precise mechanism(s) underlying natural bursts in DA neurons *in vivo* is (are) not known, our demonstration that natural and NML-induced bursts have similar characteristics allows us to generalize our findings to the physiological situation.

Our results show that low-frequency pacemaker firing is largely unaffected by XE991, presumably because the membrane potential does not reach sufficiently depolarized levels for long enough for M-channels to become substantially activated (Fig. 4A). The M-channels activate during each action potential during this firing pattern but quickly deactivate, so that no current is flowing through them at the onset of the next spike. On the contrary, during burst firing, complete deactivation is prevented by fast firing during depolarized plateaus and this allows the channels to exert their inhibitory effect under these circumstances.

In addition to its quantitative enhancement of burst firing, suppression of the M-current also alters the quality of the bursts, with a relative enrichment of very short ISIs. This effect is likely to be biologically important because it will increase the saturation of dopamine transporters at the terminals and hence sharpen the increases in the concentration of dopamine. The effect of M-current blockade on the distribution of ISIs was more spectacular in the model than in the experiments (compare Fig. S5B and D). This is probably due to the fact that the dominant repolarizing currents (other than the SK-current) after the action potential deactivate too quickly in the model. This leads to a high proportion of closely spaced action potentials.

Our results on XE991 differ from those of Sotty *et al.* (2009), who observed a significant increase in bursting in the same species when the M-current blocker was administered alone (i.e. without SK blockade). There are several possible reasons for this discrepancy. (i) Their recordings were made in the ventral tegmental area and it cannot be excluded that the density and/or topography of M-channels is different in the two areas. Moreover, the tone of excitatory and/or inhibitory afferents may also be different. This may in turn induce differences in the amount of bursting in control conditions. (ii) XE991 was administered intravenously (vs. intraperitoneally in our case) and the effect was observed at 1 and 2 mg/kg. It is probable that the brain concentrations achieved in their experiments were higher than in our intraperitoneal experiments. However, we also did not see an effect of XE991 alone in our iontophoresis experiments, even when using a higher concentration of XE991 (10 mm instead of 1 mm, not shown). (iii) The data analysis is different in the two studies. Sotty *et al.* analyzed the ‘percent changes in burst firing’, whereas we used a more conservative analysis, using the absolute value of the proportion of spikes that occur in bursts. We felt that the latter analysis is preferable because the first analysis may give too much importance to changes occurring in neurons with a small percentage of spikes in bursts (a change from 1 to 5% will be counted as a 500% change, whereas a change from 30 to 60% will be considered as 200%). However, their conclusions on the influence of retigabine on DA neurons are globally similar to ours (Hansen *et al.*, 2006).

Our results demonstrate a novel pathway for selectively altering the transient responses of DA neurons to excitatory inputs without changing their tonic low-frequency activity. Given the variety of intracellular pathways that control the M-current in central nervous system neurons (Delmas & Brown, 2005), it may offer a powerful means by which various afferent neurotransmitters can fine-tune DA transmission. One obvious candidate is acetylcholine, which blocks the M-current via M1/M3 receptors in many types of neurons. There is ample evidence for an excitatory and burst-enhancing effect of muscarinic agonists on midbrain DA neurons (Gronier & Rasmussen, 1998; Miller & Blaha, 2005). However, acetylcholine obviously has multiple effects (including inhibitory effects) on DA neuron excitability, depending on the concentration of synaptic acetylcholine and its duration of action (Fiorillo & Williams, 2000). Pharmacological modulation of the M-current should have a major impact on DA signaling that could be exploited therapeutically in the future (see Sotty *et al.*, 2009 for further discussion).

More generally, our results demonstrate that M-channels do not reduce spontaneous low-frequency firing in DA neurons. This is because of the parameters of their activation and deactivation relative to the voltage trajectory during pacemaking. However, their presence makes these neurons relatively insensitive to excitatory inputs. Therefore, modulation of this conductance may selectively control the excitability of these neurons when they are in bursting mode. This may be an advantage in some behavioral contexts.

## Abbreviations

APV: 2-amino-5-phosphonopentanoic acid
CNQX: 6-cyano-7-nitro-quinoxaline-2,3-dione
DA: dopaminergic
ISI: interspike interval
NMDA: *N*-methyl-d-aspartate
NML: *N*-methyl-laudanosine
SK: small conductance Ca^2+^ -activated K^+^
SR95531: 2-(3-carboxypropyl)-3-amino-6-(4-methoxyphenyl) pyridazinium bromide
XE991: 10,10-bis(4-pyridinylmethyl)-9(10*H*)-anthracenone dihydrochloride

## Supporting Information

Fig. S1. Effect of the i.p. administration of XE991 (3 mg/kg) on the firing rate of DA neurons in the absence of a GABA antagonist.

Fig. S2. Comparison of the interspike intervals.

Fig. S3. Relative frequency of bursts as a function of their number of spikes, and changes in spike amplitude within bursts.

Fig. S4. Comparing i.p and iontophoretic application of vehicle.

Fig. S5. Effect of M-channel blockade on the distribution of short interspike intervals.

Fig. S6. Effect of M-current blockade on a DA neuron model when the M current conductance was identical to the one reported experimentally.

Table S1: SR95531 blocks the inhibitory effect of GABA on the firing of DA neurons.

Please note: As a service to our authors and readers, this journal provides supporting information supplied by the authors. Such materials are peer-reviewed and may be re-organized for online delivery, but are not copy-edited or typeset by Wiley-Blackwell. Technical support issues arising from supporting information (other than missing files) should be addressed to the authors.
