# Supplementary material for: M-type channels selectively control bursting in rat dopaminergic neurons
Source: Eur J Neurosci. 2010 Mar;31(5):827–35. doi: 10.1111/j.1460-9568.2010.07107.x (PMC2861736; doi:10.1111/j.1460-9568.2010.07107.x)
Supplement: Supplementary file 1 [file ejn0031-0827-SD1.doc]

**Fig. S1.** A variable effect was observed in these experiments, with some cells being inhibited (N=6). **(a)** Mean frequency over time for the six cells. **(b)** Single cell frequency over time.
