# Supplementary material for: M-type channels selectively control bursting in rat dopaminergic neurons
Source: Eur J Neurosci. 2010 Mar;31(5):827–35. doi: 10.1111/j.1460-9568.2010.07107.x (PMC2861736; doi:10.1111/j.1460-9568.2010.07107.x)
Supplement: Supplementary file 2 [file ejn0031-0827-SD2.doc]

**Fig. S2.** Comparison of the interspike intervals. Both spontaneous bursts (control) and NML-induced bursts are represented. Note the similarity of all parameters in all cases. Means and SD’s are shown in order to facilitate the comparison with data of Grace and Bunney.
