# Supplementary material for: M-type channels selectively control bursting in rat dopaminergic neurons
Source: Eur J Neurosci. 2010 Mar;31(5):827–35. doi: 10.1111/j.1460-9568.2010.07107.x (PMC2861736; doi:10.1111/j.1460-9568.2010.07107.x)
Supplement: Supplementary file 3 [file ejn0031-0827-SD3.doc]

**Fig. S3.** **(a)** Relative frequency of the bursts as a function of their number of spikes. Doublets are indicated as well. **(b)** Changes in spike amplitude within bursts. One burst from our experiments (both spontaneous and NML-induced) is shown. Summary histograms for the four first ISI’s are displayed. Means and SD’s are shown in order to facilitate the comparison with data of Grace and Bunney, as in Fig. S2.
