# Supplementary material for: M-type channels selectively control bursting in rat dopaminergic neurons
Source: Eur J Neurosci. 2010 Mar;31(5):827–35. doi: 10.1111/j.1460-9568.2010.07107.x (PMC2861736; doi:10.1111/j.1460-9568.2010.07107.x)
Supplement: Supplementary file 4 [file ejn0031-0827-SD4.doc]

**Fig. S4.** **(a)** i.p. experiments (N=3). **(b)** Iontophoresis experiments (100nA) (N=5). The vehicle had no effect on the percentage of spikes in bursts in either condition.
