# Supplementary material for: M-type channels selectively control bursting in rat dopaminergic neurons
Source: Eur J Neurosci. 2010 Mar;31(5):827–35. doi: 10.1111/j.1460-9568.2010.07107.x (PMC2861736; doi:10.1111/j.1460-9568.2010.07107.x)
Supplement: Supplementary file 5 [file ejn0031-0827-SD5.doc]

**Fig. S5.** ISI’s were classified into four categories (0-40, 41-80, 81-120 and 121-160 ms, respectively). Application of XE991 significatively increased the percentage of ISI’s in the shorter intervals (**a**, intraperitoneal experiments; and **b**, iontophoresis experiments), whereas application of vehicle had no effect on this distribution **(c)**. **(d)** Simulations showed a qualitatively similar, but more robust effect. (**P* < 0.05; ***P* < 0.01; ****P* < 0.001). Note that the ordinate scale is expanded in the upper left side of each panel.
