# Supplementary material for: M-type channels selectively control bursting in rat dopaminergic neurons
Source: Eur J Neurosci. 2010 Mar;31(5):827–35. doi: 10.1111/j.1460-9568.2010.07107.x (PMC2861736; doi:10.1111/j.1460-9568.2010.07107.x)
Supplement: Supplementary file 6 [file ejn0031-0827-SD6.doc]

**Fig. S6.** Effect of M-current blockade on a DA neuron model when the M-current conductance was identical to the one reported experimentally (Koyama and Appel, 2006).
