# Supplementary material for: M-type channels selectively control bursting in rat dopaminergic neurons
Source: Eur J Neurosci. 2010 Mar;31(5):827–35. doi: 10.1111/j.1460-9568.2010.07107.x (PMC2861736; doi:10.1111/j.1460-9568.2010.07107.x)
Supplement: Supplementary file 7 [file ejn0031-0827-SD7.doc]

**Table S1.** SR95531 blocks the inhibitory effect of GABA on the firing of DA neurons
